# Microstructure Evolution of HSLA Pipeline Steels after Hot Uniaxial Compression

**DOI:** 10.3390/ma9090721

**Published:** 2016-08-24

**Authors:** Yongchang Liu, Yi Shao, Chenxi Liu, Yan Chen, Dantian Zhang

**Affiliations:** State Key Laboratory of Hydraulic Engineering Simulation and Safety, School of Materials Science & Engineering, Tianjin University, Tianjin 300072, China; ycliu@tju.edu.cn (Y.L.); shzyshy02@gmail.com (Y.S.); chenyanallana@yeah.net (Y.C.); mouselcx@gmail.com (D.Z.)

**Keywords:** HSLA piping steel, acicular ferrite, hot uniaxial compression

## Abstract

The mechanical properties of the high-strength low-alloy pipeline steels were mainly controlled by the subsequent phase transformations after rolling. The influence of hot uniaxial compression on the phase transformation of acicular ferrite was explored by viewing of the deformation degree, the deformation temperature, and the strain rate. The results show that the increase of deformation amounts raises the transformation starting and finishing temperature during the subsequent cooling and also promotes the polygonal ferrite transformation, which leads to the decrease of Vickers hardness accordingly. With the increasing of the deformation temperature, the achieved microstructure becomes coarsened and thus decreases the hardness. As the strain rate increases, the microstructure is refined and thus the hardness increases gradually; increasing the strain rate appropriately is beneficial to the refinement of the microstructure.

## 1. Introduction

Since the high-strength low-alloy (HSLA) pipeline steels were developed in the early 1970s, they have been widely used in the construction of long-distance oil and gas transportation systems, due to their good combination of strength, toughness and weldability [1,2,3]. In order to increase the transport efficiency with a higher pressure and transmission rate for long-distance pipeline, a high-grade steel with higher strength and better low-temperature toughness is needed [4,5].

The mechanical properties of pipeline steels depend on the alloy chemistry and thermo-mechanical control processing (TMCP) [6,7,8]. The latter corresponds more to the developing tendency and prospect of the modern production of the oil and gas pipeline steels. For the last 20 years, technical parameters on TMCP, such as soaking temperature, rolling temperature, finishing temperature, cooling rate and cooling interrupt temperature, have been investigated extensively [9]. Regarding the pipeline steels produced by TMCP, the microstructure may be composed of polygonal ferrite, acicular ferrite, bainitic ferrite, etc., in which the acicular ferrite–dominated microstructure is one of the most attractive candidate microstructures for pipeline steels because of its optimal combination of high strength and good toughness [10,11,12,13]. However, a small amount of “soft” polygonal ferrites also provide an important guarantee for favorable toughness, in spite of their slight handicap in strength.

This project focuses on the thermo-mechanical process in the metastable austenite field. The effects of hot uniaxial compression on the microstructure, phase transformation behaviors and micro-hardness of HSLA pipeline steel were investigated in view of the deformation degree, the deformation temperature, and the strain rate.

## 2. Experimental Details

The chemical composition of the employed material used in this project, an HSLA pipeline steel, is given in Table 1. The initial treatment was hot rolling followed by air cooling, and then the specimens were machined into cylindrical specimens with a length of 12 mm and a diameter of 8 mm for the simulation rolling experiments.

**Figure 1:**
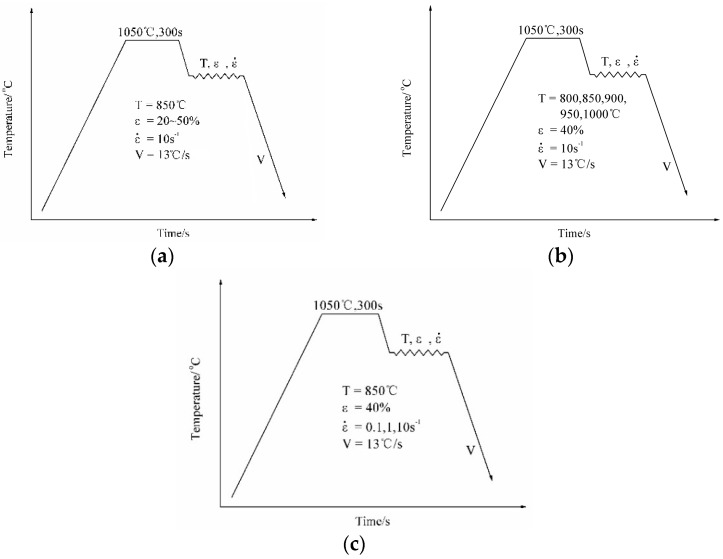
Schematic illustration of different simulation rolling procedures: (**a**) deformation degree tests; (**b**) deformation temperature tests; (**c**) strain rate tests (ε is the deformation reduction, $\overset{\cdot }{\varepsilon }$ is the strain rate, V is the cooling rate after hot uniaxial compression).

The simulation rolling experiments were performed on a Gleeble 1500 system (DSI, New York, NY, USA) equipped with a dilatometer. The simulation procedures are illustrated in Figure 1. All simulation rolling experiments were conducted under the singe pass deformation. For deformation degree experiments, the specimens were austenitized at 1050 °C for 300 s, and then deformed with various deformation degrees (20%, 30%, 40% or 50%) at 850 °C followed by cooling with a rate of 13 °C/s to room temperature (Figure 1a). For deformation temperature tests, the specimens were austenitized at 1050 °C for 300 s, and then deformed at various temperatures (800 °C, 850 °C, 900 °C, 950 °C or 1000 °C) with the deformation reduction of 40%, followed by cooling with a rate of 13 °C/s (Figure 1b). For strain rate tests, the specimens were austenitized at 1050 °C for 300 s, and then deformed with various strain rates (0.1 s^−1^, 1 s^−1^ and 10 s^−1^) followed by cooling to room temperature (Figure 1c). Due to the high thermostability of Nb carbides, the austenitizing temperature is usually selected above 1100 °C to assure the complete dissolution of precipitates. In our previous work [8], it has been found that decrease of austenitizing temperature results in increase of amount of NbC, which would promote the formation of acicular ferrite, and retard the formation of bainite. This project focuses on the formation of acicular ferrite rather than bainite. Therefore, the low austenitizing temperature was selected.

After the heat treatments, the samples were cut in half to ensure the middle of the samples can be shown for microstructural observation. Then they were mounted, polished, and etched in solution of nitric acid (4%) and ethyl alcohol. The microstructures were studied by C-35A OLYMPUS Optical Microscope (OM) (Japan). The phase transformation points were determined by the accessory dilatometer of the Gleeble 1500 thermal simulation testing system (DSI, New York, NY, USA). The starting and finishing points of transformation were determined by 5% and 95% for transformation fraction respectively. Vickers micro-hardness tests were performed using a load of 2 N.

## 3. Results and Discussion

### 3.1. Effect of Deformation Degree

**Figure 2:**
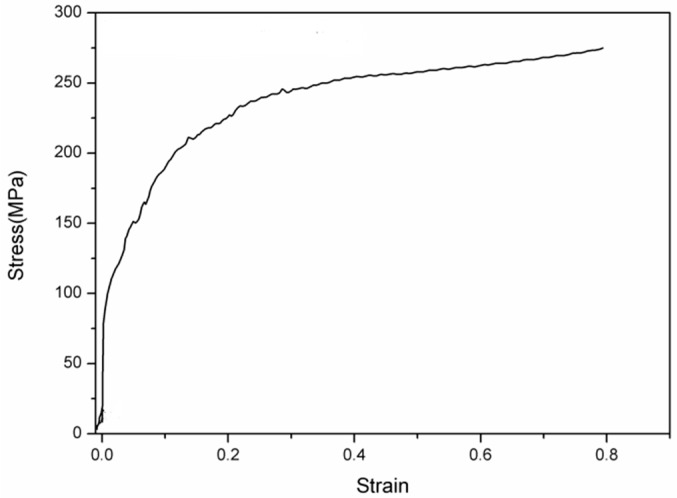
Stress-strain curve of the sample deformed at 850 °C. The strain rate for this curve is 10 s^−1^.

Figure 2 presents the stress-strain curve of the sample deformed at 850 °C. Under the condition that deformation is less than 40%, the slope of the stress-strain curve is sharply raised by the increase of the deformation reduction. It is explained that with the increase of the deformation reduction, the density of dislocations and the amount of deformation bands would increase, which results in work hardening. When the deformation reduction reaches 40%–70%, the slope of the stress-strain curve decreases gradually. This suggests that dynamic softening occurs, which may be ascribed to the recovery of dislocations and deformation bands, or formation of strain-induced ferrite.

**Figure 3:**
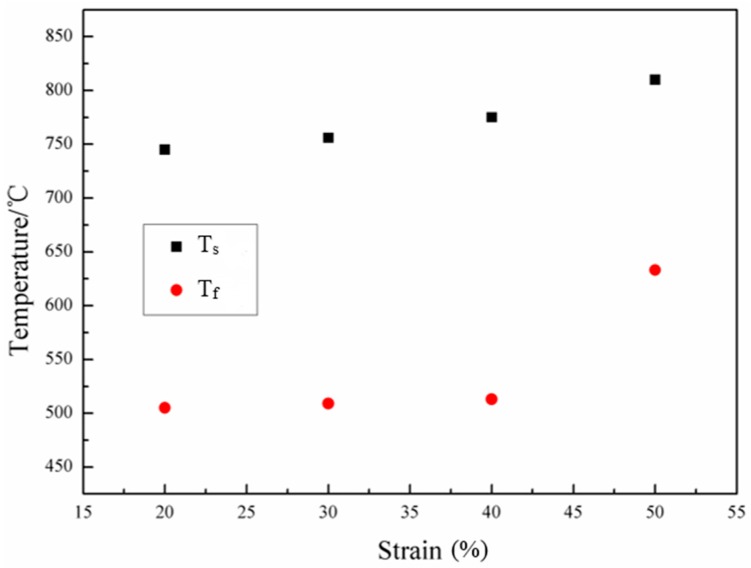
Transformation starting and finishing temperatures of the samples with different deformation amounts at 850 °C followed by continuous cooling.

The transformation starting temperature, T_s_, and finishing temperature, T_f_, of the samples during continuous cooling after deformation with various deformation reductions are shown in Figure 3. It is found that T_s_ and T_f_ are both raised as the deformation amount increases, suggesting that the process of transformation is facilitated. When the deformation reduction is higher than 50%, the increase tendency of T_s_ and T_f_ is more obvious. This is because the increase of the deformation amount results in the multiplication of dislocations and deformation bands in the metastable austenitic grain, and in the increase of cumulative strain energy, thus facilitating phase transformation during cooling after hot uniaxial compression. 

**Figure 4:**
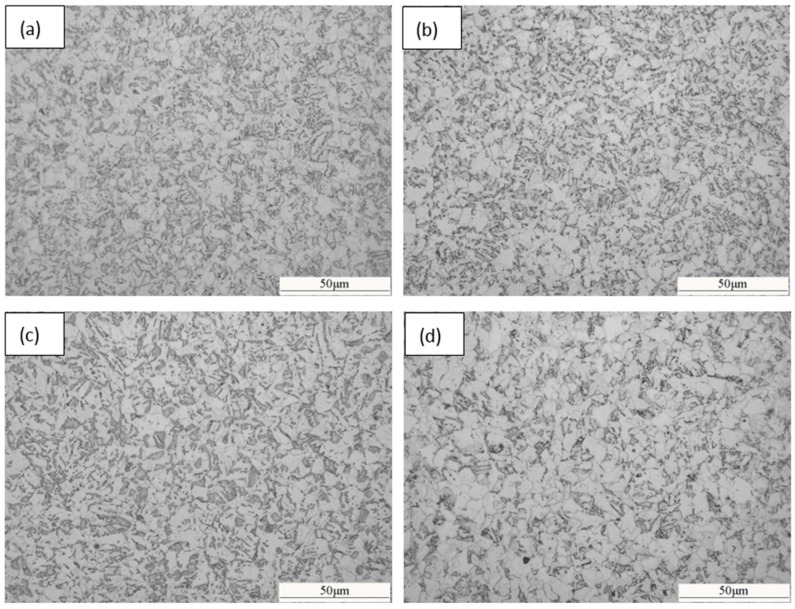
Optical micrographs of the samples with different deformation amounts at 850 °C followed by continuous cooling: (**a**) 20%; (**b**) 30%; (**c**) 40%; (**d**) 50%.

**Figure 5:**
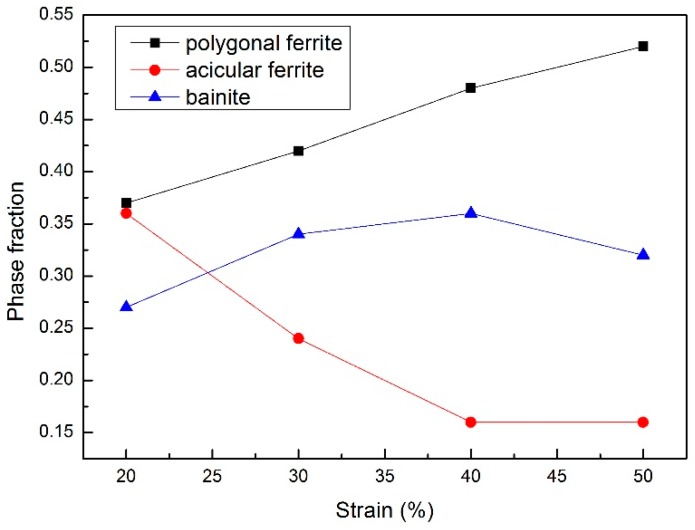
Phase fraction of polygonal ferrite, acicular ferrite and bainite for the samples with different deformation amounts at 850 °C followed by continuous cooling.

The optical micrographs of the samples with various deformation reductions (20%, 30%, 40% and 50%) are shown in Figure 4. In all samples, the microstructures are composed of equiaxed polygonal ferrite and non-equiaxed acicular ferrite and bainite. The phase fractions of polygonal ferrite, acicular ferrite and bainite determined by over five optical micrographs for each sample are presented in Figure 5. With the increase of the deformation reduction, the amount of polygonal ferrite increases, while the amount of acicular ferrite and bainite decreases. This results in an accordance with the change trend of T_s_ and T_f_ presented in Figure 3, since the temperature for the phase transformation of polygonal ferrite is higher than that of acicular ferrite. The increase of the deformation reduction enhances the amount of dislocations and formation bands, which prefer to supply the nucleation sites for polygonal ferrite.

**Figure 6:**
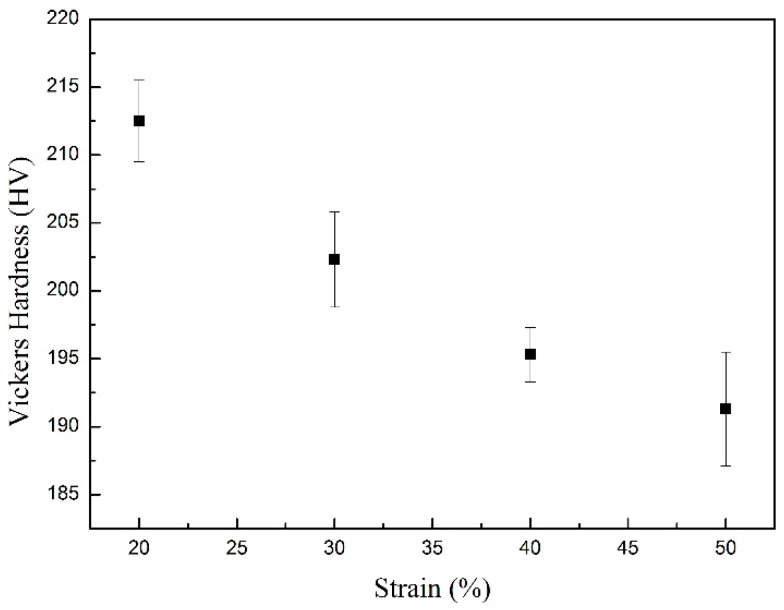
Vickers hardness of the samples with different deformation amounts at 850 °C followed by continuous cooling.

Figure 6 shows the value of the Vickers micro-hardness of the samples with various deformation reductions. It can be seen clearly that, with the increase of the deformation reduction, the Vickers hardness decreases gradually. According to the optical micrographs of the samples with different deformation reductions (see Figure 5), the increase of strain promotes the phase transformation of soft polygonal ferrite rather than that of hard acicular ferrite and bainite. Consequently, the measured value for the Vickers hardness is decreased by the increase of the deformation reduction.

### 3.2. Effect of Deformation Temperature

**Figure 7:**
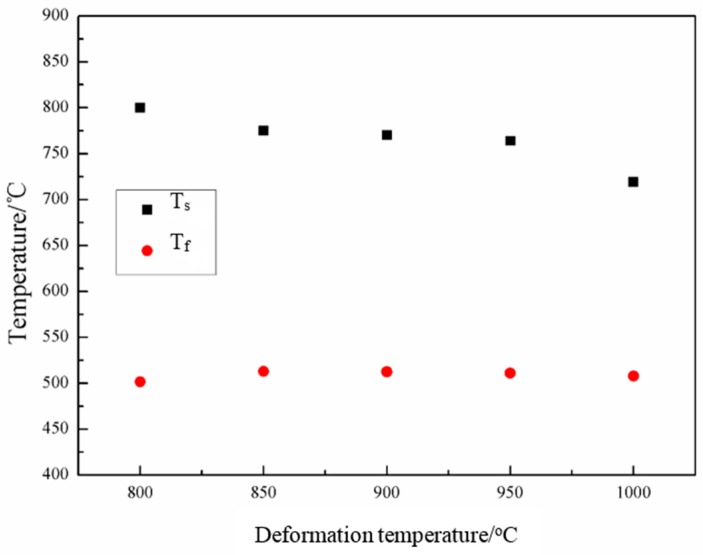
Transformation starting and finishing temperatures of the samples deformed at different temperatures.

Figure 7 presents the transformation starting temperature, T_s_, and finishing temperature, T_f_, of the samples during continuous cooling after hot uniaxial compression at various temperatures. With the increase of the deformation temperature, T_s_ decreases, whereas T_f_ remains keeps unchanged. On one hand, the rate of dynamic recovery and recrystallization is higher at the high deformation temperature, which results in the decrease of the density of dislocations and amount of deformation bands, and thus hinders the decomposition of metastable austenite during continuous cooling by decreasing the nucleation sites. On the other hand, higher deformation temperature implies that cooling to the transformation starting temperature requires more time, since the cooling rate is constant for all samples. Hence, the sample deformed at high temperature has more time for recovery and recrystallization, resulting in the decrease of nucleation sites for the subsequent phase transformation during cooling, and thus the decrease of T_s_.

**Figure 8:**
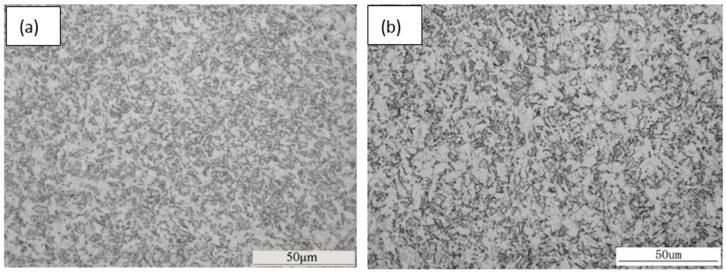

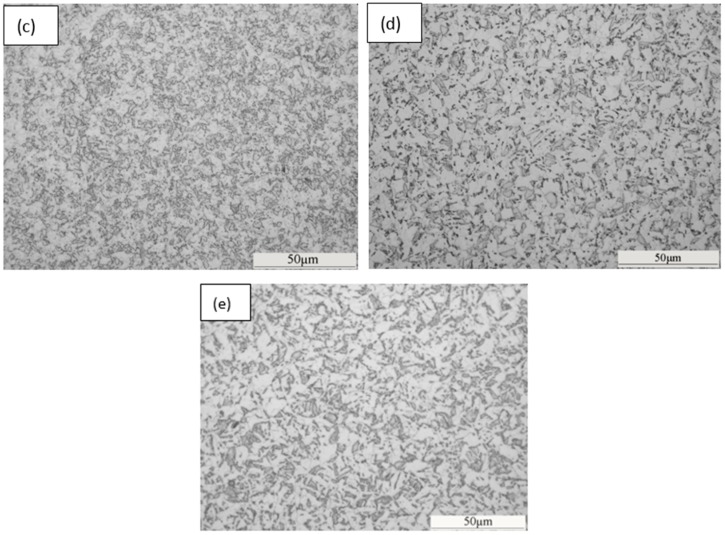
Optical micrographs of the samples deformed at different temperatures: (**a**) 800 °C; (**b**) 850 °C; (**c**) 900 °C; (**d**) 950 °C; (**e**) 1000 °C.

The optical micrographs of the samples deformed at various temperatures are shown in Figure 8. It is also found that the microstructures in all samples are a mixture of polygonal ferrite and acicular ferrite. The increase of the deformation temperature results in the microstructure coarsening. A higher deformation temperature leads to a higher rate for the recovery of dislocations and deformation bands, and thus decreases the nucleation sites. As a result, the grain size is higher in the samples deformed at the higher temperature. Besides, the ultra-fine grains with continuous distribution can be observed in the sample deformed at 800 °C (see Figure 8a). These ultra-fine grains are supposed to be strain-induced ferrite during hot uniaxial compression. It has been recognized that a relatively low deformation temperature leads to dynamic strain-induced transformation (DSIT) [14,15,16]. It is generally acknowledged that severe deformation near the A_r3_ temperature results in the formation of the ultra-fine ferrite grains, namely DSIT. The strain-induced ferrite grains form on the prior austenite grain boundaries at first, and then grow into the austenite grains. The increase of the deformation reduction can promote DSIT. The starting temperature for transformation without deformation is determined by the dilatometer measurement of 767 °C, which is closed to that for the sample deformed at 800 °C. According to Figure 6, the transformation starting temperature is 800 °C in the sample deformed at 800 °C. This also gives solid evidence for DSIT.

**Figure 9:**
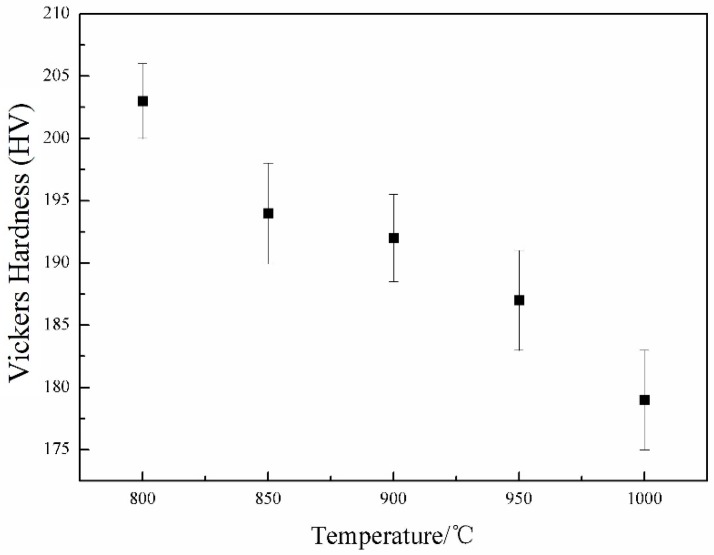
Vickers hardness of the samples deformed at different temperatures.

Figure 9 presents the Vickers micro-hardness of the samples with various deformation temperatures. It can be seen that the increase of the deformation temperature results in the decrease of the Vickers hardness. According to the microstructural observation (Figure 8), coarse grains are found in the samples deformed at higher temperatures, which has a harmful influence on hardness. Besides, a relatively high deformation temperature decreases the density of dislocations and the amount of deformation bands, which leads to the decrease of hardness.

### 3.3. Effect of Strain Rate

**Figure 10:**
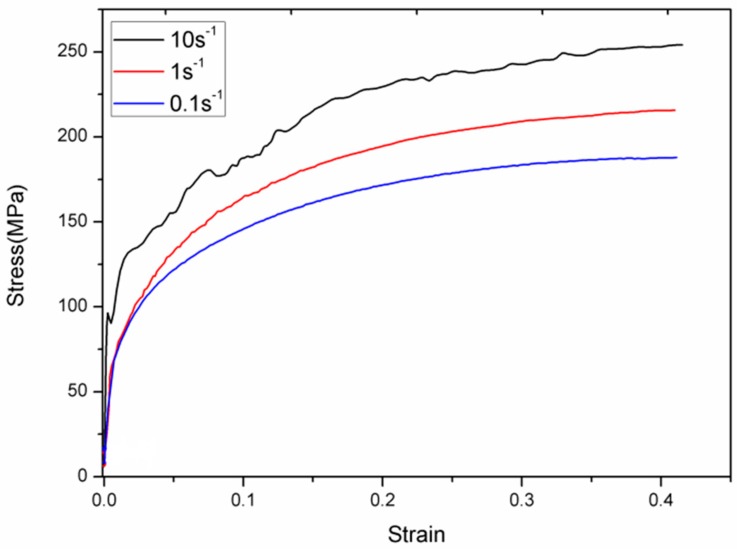
Stress-strain curves of the specimens under different deformation rates at 850 °C.

**Figure 11:**
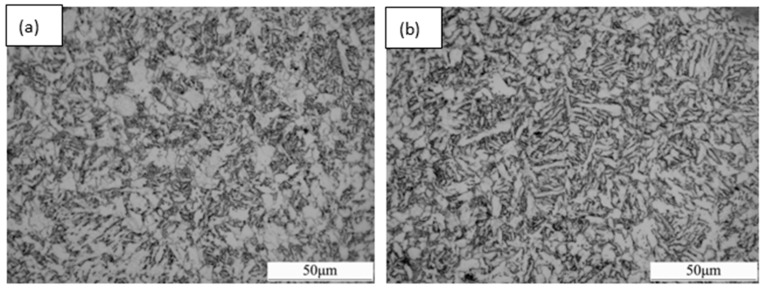

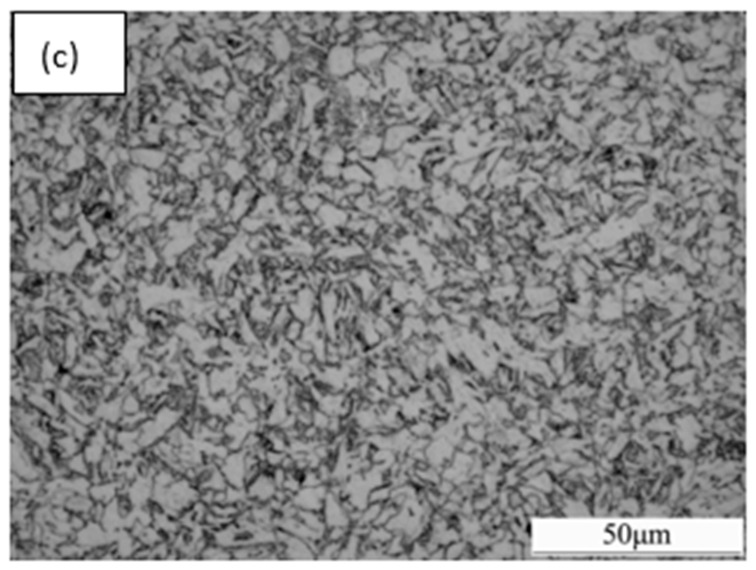
Optical micrographs of the samples with different deformation rates at 850 °C: (**a**) 0.1 s^−1^; (**b**) 1 s^−1^; (**c**) 10 s^−1^.

Stress-strain curves of the samples during hot uniaxial compression with various strain rates are presented in Figure 10. With the increase of the strain rate, the upward trend of the curves is more obvious, and the value of the stress is higher, corresponding to the same value for the strain. Under the high strain rate, dislocations and deformation bands resulting from the deformation do not have enough time for recovery, and need higher stress to reach the same value as the strain. The three stress-strain curves are all in the rising trend under the condition that the strain values are up to 0.4, showing the typical work-hardening behaviors. Furthermore, it should be noticed that the curve at the strain rate of 10 s^−1^ has evident fluctuations (see Figure 11). This may be due to the so-called Portevin-Le Chȃtelier (PLC) effect, which results from dynamic strain-aging [17]. The origin of the PLC effect is associated with the interaction between the motion of mobile dislocations and the diffusion of interstitial atoms as C or N toward the dislocation cores. It has been reported that a higher strain rate results in the PLC effect occurring at a higher temperature [17]. Besides, the dynamic transformation of deformed austenite may also be the reason for the appearance of the serrate-shaped curve [18]. The dynamic transformation would also bring about the softening of austenite.

Figure 11 presents the optical micrographs of the samples with different strain rates. It can be seen that the microstructure of all samples is composed of acicular ferrite and a small amount of polygonal ferrite. With the increase of the strain rate, the grain size decreases. The relatively low strain rate provides enough time for the recovery of dislocations and deformation bands, and thus decreases the nucleation sites for subsequent polygonal ferrite and acicular ferrite transformation, resulting in the formation of coarse grains. On the contrary, the relatively high strain rate blocks the recovery of dislocations and deformation bands, refining the grains by increasing nucleation sites. It is suggested that the increase of the strain rate in the metastable austenite field (such as 850 °C in this case) would enhance the mechanical properties by refining the grains.

**Figure 12:**
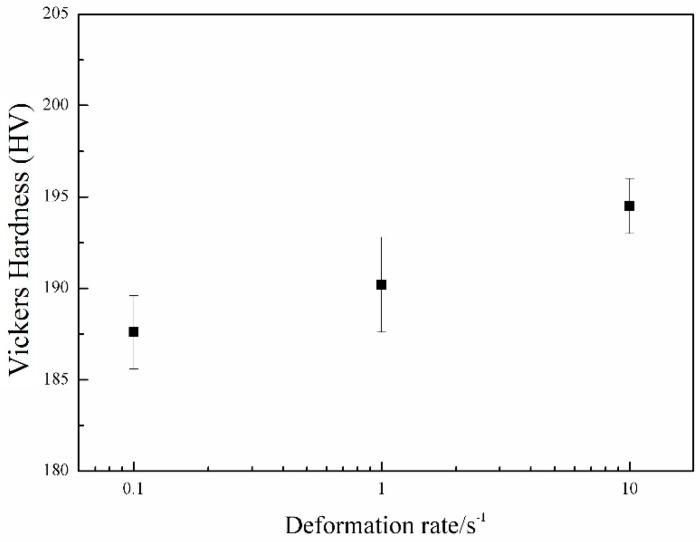
Vickers hardness of the samples with different deformation rate at 850 °C.

The Vickers micro-hardness results of the samples with various strain rates are shown in Figure 12. With the increase of the strain rate, the Vickers hardness is increased gradually. This corresponds to the microstructural observations. The higher strain rate results in finer grains, and thus increases hardness.

## 4. Conclusions

In this paper, the effects of hot uniaxial compression on the microstructure, phase transformation behaviors and mechanical properties in view of the deformation degree, deformation temperature and strain rate were investigated. The conclusions are as follows:

i. The increase of the deformation reduction results in the increase of the transformation starting and finishing temperature for polygonal ferrite and acicular ferrite, due to the multiplication of dislocations and deformation bands providing more nucleation sites. The increase of the deformation reduction promotes the formation of polygonal ferrite and hinders that of acicular ferrite, and thus decreases the Vickers hardness.

ii. The increase of the deformation temperature leads to the decrease of the transformation starting temperature, due to dynamic recovery at high temperature and enough recovery time during cooling. The increase of the deformation temperature results in coarse grains, and thus decreases the Vickers hardness. Deformation at 800 °C leads to dynamic strain-induced transformation.

iii. A higher strain rate results in a finer microstructure and higher Vickers hardness. The relatively low strain rate provides enough time for the recovery of dislocations and deformation bands, and thus decreases the nucleation sites, resulting in the formation of coarse grains.

## Figures and Tables

**Table 1:** Chemical composition of the tested HSLA pipeline steel (wt. %).

C	Si	Mn	Ni	Cr	Mo	Cu	Al	V	Nb	Ti
0.09	0.29	1.29	0.15	0.06	0.18	0.13	0.034	0.05	0.03	0.001
